# Critical success factors for the successful initiation of Lean in public hospitals in KwaZulu-Natal: a factor analysis and structural equation modelling study

**DOI:** 10.1186/s12960-019-0405-1

**Published:** 2019-08-23

**Authors:** Logandran Naidoo, Ziska Fields

**Affiliations:** 1 University of KwaZulu-Natal, Pietermaritzburg, South Africa; 2 University of Johannesburg, Johannesburg, South Africa

## Abstract

**Background:**

Lean thinking is one of several operations-management techniques which have yet to be fully embraced in the South African health care sector. In most health care managers’ service delivery mandates, *what* needs to be done might be known, but it is *how* it should be done which might be alien to most managers. In order to recognise the “how”, one needs to know the critical success factors for Lean initiation.

**Methods:**

The research took the form of an observational descriptive study with quantitative methods. The objectives were to identify the key variables for the successful initiation of Lean and then to conduct factor analysis and structural equation modelling (SEM) on these variables leading to the identification of critical success factors (CSFs) for Lean initiation. Simple random sampling was applied to select the participants from various categories of 500 senior managers across 73 KwaZulu-Natal (KZN) public hospitals. The sample size was 218, with a response rate of 96.8% (*n* = 211). For the purpose of identifying key variables for the successful initiation of Lean and then of conducting factor analysis and SEM on these variables, a self-administered, structured questionnaire was used. Data were reduced using exploratory factor analysis (EFA) to identify latent constructs. Confirmatory factor analysis (CFA) was used to determine the reliability and validity of these factors. Structural equation modelling (SEM) fit indices were then applied to assess acceptability of the measurement model.

**Results:**

Certain variables were eliminated during EFA if they cross-loaded onto more than one factor, since this caused discriminant validity problems. In addition, if variables loaded weakly onto a factor, they were not retained. Three critical success factors (CSFs) were identified in this study: strategic leadership and organisational attitude; integration of Lean elements, tools, and techniques; and basic stability in operational processes. All reliability and validity conditions have been met (RMSEA = 0.085; CFI = 0.956 and *χ*^2^/df = 2.513), consequently rendering the model reliable and valid.

**Conclusion:**

None of the three CSFs can be viewed in isolation, as they all have significance at different dimensions of capability within the organisation. The use of these CSFs and the context, content, application, and outcome of Lean should be viewed in light of the organisation’s strategic, technical, structural, and cultural environment. Further research in the effectiveness of these CSFs for the rollout of Lean in South African hospitals would be of benefit to the Lean body of knowledge.

## Introduction

The volatile environment for health care delivery in South Africa, due to its quadruple burden of communicable, non-communicable, perinatal and maternal, and injury-related disorders with generally poor health outcomes, compels health care managers to adopt contemporary management approaches shown to be effective in resource-constrained environments [1–3]. Lean thinking is a philosophy involving proven operations practices and techniques that improve the quality and efficiency of production and service delivery by enhancing operational flow and diminishing wasteful activity in an organisation [4]. However, the question of what critical success factors will predict the success of Lean initiation in public hospitals remains unanswered and receives little attention in the literature describing the South African health care context.

## Background

Lean thinking is one of several operations management techniques which have yet to be fully embraced in the South African health care arena [5–7]. The primary focus of Lean is on reducing waste, synchronising flows, and managing variability in (process) flows [8]. Lean methodology comprises five fundamental tenets [9, 10]:

- *To specify what is of value* to the end-user (the patient)
- *To identify the value stream* in a workflow process
- *To make the value stream flow* by re-engineering process steps and eliminating bottlenecks
- *To create pull* down the value-stream which signals when upstream activities can begin
- *To pursue perfection* through continuous improvement

Lean classifies activities in a value stream into three categories: (1) value-added work; (2) type 1 non–value-added work, which is necessary but does not add value from the standpoint of the patient; and (3) type 2 non-value-added work (waste or *muda*) which does not add any value to the patient from any perspective and should therefore be eliminated [4].

Lean has been revolutionising manufacturing and service industries globally for many years and is endorsed as “creat[ing] a balance between quality and finance by developing the most efficient and effective method of providing value to the customer” [4]. Faull posits that the application of Lean to health care has begun in earnest, mainly in the United States of America, the United Kingdom, and Australia [11]. However, the current application of Lean in Southern African health care lacks coherence, despite its increasing prevalence in health institutions [5, 7, 12].

Substantial resource constraints in the face of increasing demands on the health care system are cited as important factors underlying mismanagement in public sectors such as health [13]. In order to sustainably recuperate the delivery of health care in the context of the current local challenges of limited resources and poor prospects for economic growth, efforts must be made to create an improved health care management based on a philosophy of “doing better with less” [3]. It is therefore imperative that a fundamental shift in management philosophy be established to create a platform that nurtures inspiration and encourages productivity through efficiency. In most health care managers’ service delivery mandates, *what* needs to be done may be known, but *how* it should be done remains alien to most managers. To recognise the “*how*”, the critical success factors for Lean initiation must be acknowledged and emphasised.

Critical success factors (CSFs) can be defined as “the limited number of areas in which results, if they are satisfactory, will ensure successful competitive performance for the organisation” [14]. It has been posited that research results show both successful and unsuccessful Lean implementation, indicating that CSFs for its initiation must be recognised [15]. Further observations reveal that empirical literature to “evidence *how* Lean implementation is operationalised in healthcare over and above a few isolated case studies that often describe a successful, but isolated project” is lacking [16].

International studies have shown that there are striking similarities in the systemic application of Lean in three settings, namely, the United States of America, Australia, and the United Kingdom [17–19]. These similarities include starting from a crisis standpoint, leadership commitment, commitment to organisational change, the use of rapid improvement events or *kaizen* events, structured problem identification and solving skills, training of staff on Lean, and the rigorous application of Lean tools. Organisational readiness factors such as a clear understanding of the system view, patient perception, the application of information, and engaging employees are also cited as key variables for preparing for Lean implementation [20].

In the South African health sector, however, there is a paucity of research on CSFs for Lean initiation. Some researchers merely describe the challenges and barriers of Lean implementation in the South African health sector, for example, the variability of processes and patient flow, a lack of understanding of Lean, poor communication and leadership, difficulty in defining waste, and the challenge of defining value from the patient’s perspective [11, 21, 22]. Simply identifying the challenges of Lean, however, does not necessarily translate into identifying CSFs.

In an influential study involving a systematic review of 33 articles on PubMed, Web of Science, and Business Source Premier, four different change mechanisms were identified as positive results yielded through Lean. Outlined in all 33 articles, these change mechanisms were understanding processes, planning and organising for effectiveness and efficiency, increasing awareness and process reliability, and collaboration amongst staff to solve problems systematically [23].

From a seminal literature review of 177 research papers dating from 2000 to 2015—conducted across several elements of health care operations management, including service quality, service operations strategy, service scheduling, service performance, and frontline employees [24]—it is clear that more health care operations management research is required in developing and underdeveloped countries due to the unique challenges experienced in these nations in comparison to developed nations. The literature review revealed that a large proportion of empirical studies have been conducted only in developed nations [24], a lack of balance that needs to be rectified.

In the reviewed literature, apart from the elaborate descriptions of the challenges and success factors for Lean implementation, mainly in non-health care organisations, it is clear that there is a dearth of studies in the health care sector in South Africa. None of the reviewed literature reveals any proposals or recommendations for the identification or application of CSFs for Lean in health care in South African hospitals.

## Methods

The research was centred on a positivist paradigm and took the form of an observational, descriptive study with quantitative methods. The primary aim of the study was to develop a Lean Success Predictor for Rapid Initiation Tool (Lean-SPRInT) for the implementation of Lean in public hospitals across KwaZulu-Natal, South Africa. Two of the study’s objectives, the results of which are described in this article, were to identify the key variables for the successful initiation of Lean and then to conduct factor analysis and SEM on these variables, thus leading to the identification of CSFs for Lean initiation.

Although they remain beyond the scope and content of this article, other objectives of the study included uncovering the knowledge and experience of Lean amongst senior health care managers in KwaZulu-Natal, South Africa, and utilising the identified CSFs to develop a Lean Success Predictor for Rapid Initiation Tool (Lean SPRInT) for the successful initiation of Lean.

**Fig. 1:**
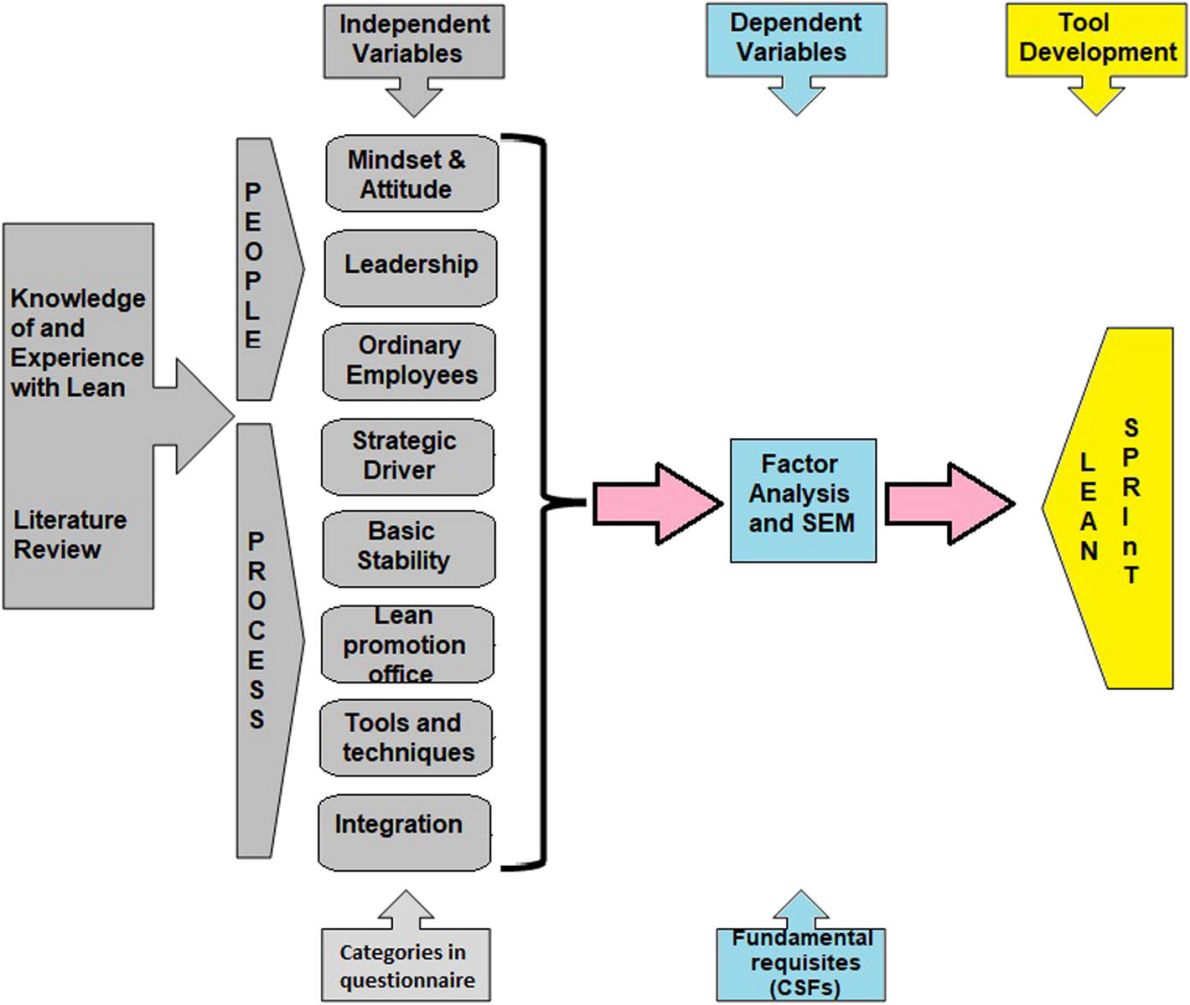
A conceptual framework for the current study (source: author developed)

From an extensive literature review, Vermaak ([25], p., 183) established that the independent variables considered as CSFs for Lean implementation in the manufacturing sector can be classified under 8 categories: mindset and attitude, leadership, ordinary employees, strategic driver, basic stability, Lean promotion office, tools and techniques, and integration [25]. With permission, the researcher (and authors of this article) utilised these categories and independent variables in the data collection tool, subjected them to Likert scale ratings by senior health care managers, and conducted factor analysis to identify the CSFs (dependent variables) which would be incorporated into the Lean SPRInT. This process is reflected in the conceptual framework (Fig. 1).

### Study site, target population, and sampling

The research was conducted in public health facilities (Table 1) within the province of KwaZulu-Natal (KZN), South Africa, which is the second most densely inhabited province out of nine in the country [26]. KwaZulu-Natal is located in the eastern coastal region of South Africa. Its population comprised approximately 11 074 784 citizens in 2017, according to 2016 and 2017 mid-year population estimates from Stats SA [27].

**Table 1:** Public health facilities in KwaZulu-Natal [27]

Health district	Primary health care (PHC) facilities			Hospitals (public + state aided)							
	Fixed clinics	Community health centres	Total PHC facilities	District	Regional	Tertiary	Central	Specialised tuberculosis	Specialised psychiatric	Chronic/sub-acute	Total hospitals
Ugu	51	2	53	3	1	0	0	1	0	0	5
Umgungundlovu	50	3	53	2	1	1	0	2	3	0	9
Uthukela	36	1	37	2	1	0	0	0	0	0	3
Umzinyathi	53	1	54	4	0	0	0	0	0	0	4
Amajuba	25	1	26	1	2	0	0	0	0	0	3
Zululand	71	1	72	5	0	0	0	1 + 2	1	0	9
Umkhanyakude	57	0	57	5	0	0	0	0	0	0	5
King Cetshwayo	63	1	64	6	1	1	0	0	0	0	8
iLembe	34	2	36	3	1	0	0	0	0	0	4
Harry Gwala	39	1	40	4	0	0	0	1	1	0	6
eThekwini	119	8	127	3 + 1	6	1	1	2	1	2	17
Total	598	21	619	39	13	3	1	9	6	2	73

Non-probability, purposive type sampling was used in order to focus the inquiry based on the particular characteristics of targeted senior managers. The target population included approximately 500 senior managers (some of them acting managers), based in 73 public hospitals in the province, consisting of the following ranks: hospital executive managers, assistant managers, operational or unit managers, and managers of clinical departments. Simple, random sampling was applied to select the participants from these categories of senior managers.

For exploratory factor analysis, it is proposed that a reliable sample size is one which contains *n* subjects for every test item (*p*), where *n* equals at least 5 [12]. Some factor analysis experts argue that the *n*:*p* ratio should be at least 3 to 6 per test item whilst others recommend a minimum of 5 per test item [12, 28, 29]. Table 2 shows that a larger proportion (a cumulative percentage of 63.2%) of studies use between 2 and 10 subjects per test item. There is no hard and fast rule to the sample size for exploratory factor analysis. In this study, there are 32 test items in the questionnaire.

**Table 2:** Factor analysis sample sizes in current practice [12]

Subject to item ratio	% of studies	Cumulative %
2:1 or less	14.7	14.7
> 2:1, ≤ 5:1	25.8	40.5
> 5:1, ≤ 10:1	22.7	63.2
> 10:1, ≤ 20:1	15.4	78.6
> 20:1, ≤ 100:1	18.4	97.0
> 100:1	3.0	100.0

As a result, for reliable factor analysis, a sample size of at least 192 (based on a subject to item ratio of 6:1) was required. The planned sample size of senior managers, considering a 5% margin of error and a 95% confidence interval, was 218. The response rate was 96.8% (*n* = 211).

The sample size can be regarded as acceptable if the communalities are high (squared multiple correlation > 0.6) and factors relatively few in number; MacCallum et al. [30] explain that with the above conditions, the “investigator can be confident that obtained factors represent a close match to population factors even with moderate to small sample sizes” since the *n*:*p* ratio recommendations above may not be invariant across studies. The communality of a variable (frequently estimated by the squared multiple correlation) can be defined as “the portion of the variance of that variable that is accounted for by the common factors” [30]. The authors further recommend post hoc judgement of the adequacy of the sample size used for factor analysis, by examining communalities and number of factors. Consequently, in terms of MacCallum et al.’s [30] proposition, factor analysis in this study shows that communalities are high (mostly above 0.6) and factors few in number (3 factors); hence, the sample of 211 is reliable.

### Inclusion and exclusion criteria

Any of the executive or senior managers mentioned above who declined participation in the study were excluded. All senior managers from the categories described above, based in 73 public hospitals in KZN, will be included in the sampling frame, irrespective of their duration in the post and whether they are acting in a vacant position or not.

### Data collection

For the purpose of identifying key variables for the successful initiation of Lean, and then to conduct factor analysis and SEM on these variables, a self-administered, structured questionnaire with categorical and variable Likert-scale questions was used for data collection. These were distributed to 218 randomly selected senior managers across the public hospitals of KZN.

### Data analysis

Statistical analyses were carried out using the SPSS® software package. Factorability of the variables was determined by measures of sampling adequacy: Kaiser-Meyer-Olkin (KMO) and Bartlett’s test of sphericity. Cronbach’s alpha was used to determine the internal consistency of the test items contained in the questionnaire, looking particularly for unidimensionality (homogeneity) of items measuring latent constructs [31]. Cronbach’s alpha generally > 0.7 was considered acceptable [31].

Data were reduced using exploratory factor analysis (EFA) to identify latent constructs. Confirmatory factor analysis (CFA) was used to determine the reliability and validity (both convergent and discriminant) of these factors. Structural equation modelling (SEM) fit indices were then applied to assess acceptability of the measurement model.

## Results

### Response rate and general characteristics of respondents

**Fig. 2:**
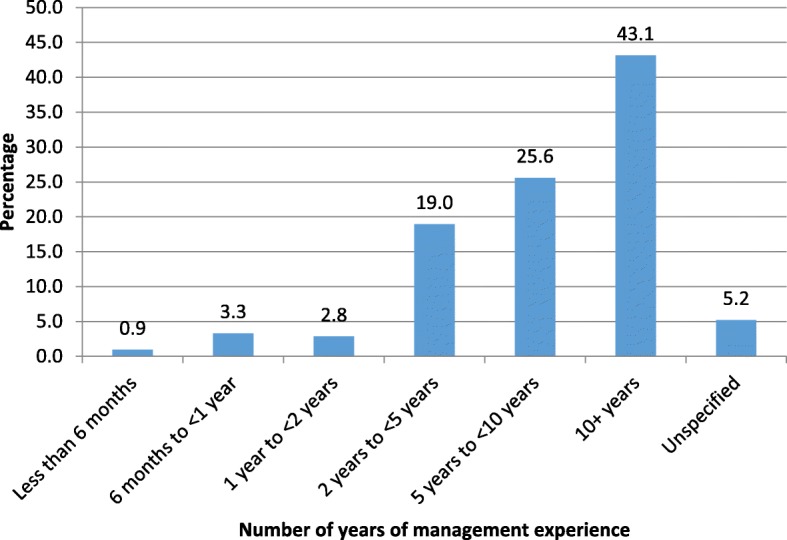
Respondents’ management experience in years

A total of 211 responses were received (96.8% response rate). Most of the respondents (43.1%) possessed more than 10 years of management experience, followed by a mediocre proportion (25.6%) of them having 5 to 10 years’ management experience (Fig. 2). A smaller proportion (19.0%) possessed 2 to 5 years of management experience.

### Exploratory factor analysis and confirmatory factor analysis

Initial EFA produced structures that could not yield factors which showed discriminant validity. This was as a result of the very high correlations between some of the factors. Some variables were eliminated during EFA if they cross-loaded onto more than one factor since this causes discriminant validity problems. In addition, if variables loaded weakly onto a factor, they were not retained.

**Fig. 3:**
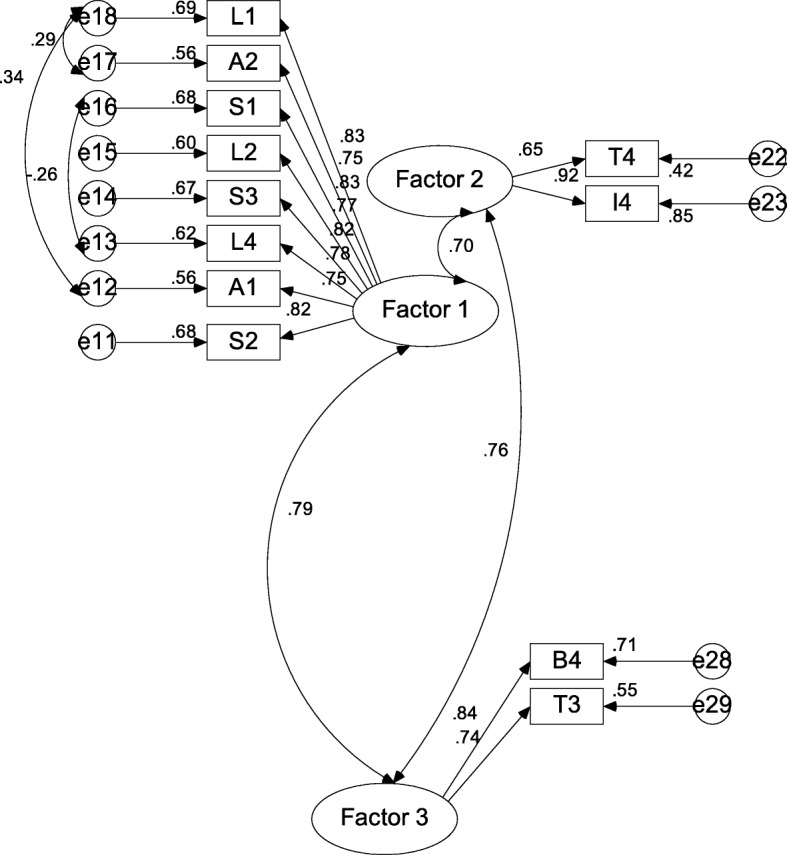
Depiction of confirmatory factor analysis results

The CFA measurement model of the factors showing correlations is provided below, along with the tables of output for the values shown in Fig. 3.

The standardised regression weights (SRW) or loadings of the test items or variables onto the factors are reflected in Table 3.

**Table 3:** Standardised regression weights of test items

Test item code		Factor label	SRW
A1	←	Factor 1	0.75 0
L4	←	Factor 1	0.78 4
S3	←	Factor 1	0.81 9
L2	←	Factor 1	0.77 4
S1	←	Factor 1	0.82 6
A2	←	Factor 1	0.74 5
L1	←	Factor 1	0.83 2
S2	←	Factor 1	0.82 4
T4	←	Factor 2	0.65 2
I4	←	Factor 2	0.92 4
B4	←	Factor 3	0.84 2
T3	←	Factor 3	0.74 3

Correlations between the factors and squared multiple correlations (communalities or the amount of variance in the observed test item or variable that the factor or construct explains) are reflected in Tables 4 and 5, respectively.

**Table 4:** Correlations between factors

Factor label		Factor label	Correlation
Factor 1	↔	Factor 2	0.69 6
Factor 3	↔	Factor 2	0.75 9
Factor 3	↔	Factor 1	0.78 7

**Table 5:** Squared multiple correlations of test items

Test item code	Squared multiple correlation (communality)
S2	0.67 9
T3	0.55 1
B4	0.70 9
I4	0.85 4
T4	0.42 5
L1	0.69 3
A2	0.55 5
S1	0.68 2
L2	0.59 9
S3	0.67 1
L4	0.61 5
A1	0.56 2

Derived from EFA and CFA, three critical success factors have been identified in this study for the successful initiation of Lean in public hospitals (Table 6)

**Table 6:** Critical success factors for Lean initiation in hospitals

Critical success factors	Elements (taken from test items)
CSF1: Strategic leadership and organisational attitude	L1: Leadership at all levels in the organisation must drive, live, and demonstrate Lean behaviour.
	A2: An organisation implementing Lean must face and embrace the various attitudinal aspects of Lean.
	S1: Lean philosophy and principles must be reflected in the organisation’s business strategy.
	L2: Lean leadership leads to Lean thinking.
	S3: Lean implementation must be driven as a high priority strategic business initiative.
	L4: The difference between Lean success and failure starts with leadership.
	A1: The mindset and attitude or behaviour of people is fundamental to Lean success.
	S2: There must be a clear link between the organisation goals, key objectives, and Lean activities.
CSF2: Integration of Lean elements, tools, and techniques	I4: The organisation must use all the goals, methods, techniques, and foundation elements of Lean in combination
	T4: The application of Lean tools and techniques will ensure Lean success.
CSF3: Basic stability in operational processes	T3: It is important to understand the organisation’s processes and only apply the Lean tools and techniques applicable to that specific process type.
	B4: Stability in operating systems is a pre-requisite for Lean transformation.

### Structural equation modelling fit indices

To assess whether this was an acceptable CFA measurement model, SEM non-centrality-based fit indices were assessed (Table 7).

**Table 7:** Structural Equation Modelling (SEM) non-centrality based fit indices

SEM fit index	Recommended cut-off value	Value for this model
Root mean square error of approximation (RMSEA)	< 0.08 [32, 33] Some authors allow < 0.10 for a fair/mediocre fit [34]	0.08 5
Comparative fit index (CFI)	≥ 0.95 [35, 36]	0.95 6
Relative or normed chi-square (*χ*^2^/df)	< 5 [37]	2.51 3

### Reliability and validity of model

The following conditions are required for reliability and validity of the model:

- Reliability: composite reliability (CR) > 0.7 and loadings on factors > 0.5
- Convergent validity: CR > average variance extracted (AVE) and AVE > 0.5
- Discriminant validity: AVE > squared correlations

For this model, Table 8 shows the values of the indices used to assess for reliability and validity, based on the conditions specified above. Diagonals represent AVE and Alpha represents Cronbach’s alpha reliability measure. Off diagonals represent squared correlations (also known as shared variance).

**Table 8:** Squared correlations, composite reliability, average variance extracted, and Cronbach’s alpha for current model

	Squared correlations				
Construct	F1	F2	F3	CR	Alpha
F1	0.63 2			0.93 2	0.93 4
F2	0.48 4	0.63 9		0.77 5	0.71 5
F3	0.61 9	0.57 6	0.63 1	0.77 3	0.76 3

**Table 9:** Corresponding critical success factors for Lean identified in other industries

Context and researchers	CSFs surveyed from literature (corresponding CSFs identified in current study indicated in parentheses)
CSFs relevant to measuring the degree of success of Lean implementation in information technology support services [15]	Management leadership (CSF1); management support (CSF1); top management commitment (CSF1); organisational culture; communication; training and skill building; financial capability; measurement framework
Implementation of Lean manufacturing within SMEs [41]	Leadership and management (CSF1); financial capability; skills and expertise and organisational culture
Enablers and inhibitors during the implementation of Lean in a Mexican public service organisation [42]	Commitment to and wish for improvement (CSF1); clear resolve to improve; focus on the simple and practical; active leadership (CSF1); outcome or stakeholder-oriented service; holistic and transversal thinking; establishing a system for measuring service process performance; effective implementation of best human resource management practices
Success factors identified during two Lean implementation projects within the same company: a global manufacturer of food processing machines and equipment [43]	Management commitment to, and involvement in, the Lean effort (CSF1); employee autonomy to make decisions regarding business process changes; information transparency of Lean goals; evidence of initial performance improvements and long-term sustainability of Lean efforts
A secondary review of research literature of key factors of success in the management of the synchronised production system (SPS) implementation process [44]	Business plan and vision; top-management support (including funding) (CSF1); project management (including project champion and teamwork and composition); change management, organisational culture; effective communication, education and training, knowledge transfer, knowledge management (including skills and expertise); organisational structure; monitoring and evaluation of performance: performance measurements
Critical success factors within SMEs implementing lean [45]	Management involvement and commitment (CSF1); communication; link quality improvement to employee; culture change; education and training; link quality improvement to customer; project selection; link quality improvement to business; link quality improvement to supplier; project management skill; organisation infrastructure; vision and plan (CSF1); information technology and innovation.
Ten CSFs for software industries from a pilot study [46]	Leadership engagement and uncompromising commitment of top management (CSF1), supporting OI, cultural change, Lean training, linking Lean to business strategy, accountability, customer involvement, understanding of Lean methodology (CSF2 and CSF3), project management, project prioritisation, and selection
Four essentials for successful implementation of a Lean programme [39]	Belief that the new programme will work; commitment for implementing it from managers (CSF1); involvement of the whole organisation—employees and resources; patience and long-term view of the results

All reliability and validity conditions have been met, thus rendering the model reliable and valid.

### Critical success factors identified in this study

Three CSFs have been identified in this study for the successful initiation of Lean in public hospitals: strategic leadership and organisational attitude; integration of Lean elements, tools, and techniques; and basic stability in operational processes. Each CSF consists of elements which itemise the factor (Table 6). The elements of each factor provide a brief statement of the key requirements for health care managers to consider prior to the initiation of Lean in public hospitals, the absence of which may impede successful Lean rollout.

## Discussion

For various manufacturing organisations, it appears that clear organisational goals, values, and vision and the communication thereof; emphasis on leadership and commitment; and resource capabilities are acknowledged as common CSFs for Lean [38–40]. A large number of studies in non-healthcare industries on the CSFs for Lean implementation describe several common CSFs (Table 9). However, only one of the three identified CSFs (CSF1: strategic leadership and organisational attitude) closely resembles some of those described in the majority of studies reviewed for non-healthcare industries (Table 9).

Management leadership and incorporation of Lean in the organisation as a strategic driver is paramount as a Lean CSF in research literature, and the findings of this study in the KZN public hospitals corroborate this CSF as instrumental also within the health care industry [47–51]. The other two CSFs (integration of Lean elements, tools, and techniques and basic stability in operational processes) appear peculiar to several of the non-healthcare industries as enablers or Lean implementation success indicators, but this study shows that they are nevertheless identified as critical in KZN public hospitals.

CSF2 and CSF3 are fundamental to the 5 Lean principles (specify value, identify the value stream, create flow, allow pull, and pursue perfection) [9]. The application of these principles justifies these two CSFs as integral rudiments for successful Lean initiation in the healthcare industry. Hospitals are rarely based on a typical assembly line structure; they are person-orientated operations with various “patient processing” service nodes in which unique and multi-faceted events occur. This would emphasise the need for the integration of Lean elements, tools, and techniques, and the requirement of basic stability in some of the value stream processes in order for Lean to be applied. One may ask, what is Lean without its signature elements, tools, techniques, and processes of flow in the value stream?

More importantly, each of the three CSFs cannot be viewed in isolation, as they all have significance in different dimensions of capability within the organisation, characteristically represented by Andersen et al.’s [51] framework of Lean facilitators (Table 10).

**Table 10:** Andersen et al.’s framework of Lean facilitators identified in literature reviews from 2000 to 2012 [51]

Dimensions of capability	Domain of the intervention			
	Context	Content	Application	Outcomes
	Situation and organisation	Characteristics of the intervention	Local delivery process	Results and maintenance
*Cultural* Underlying beliefs, values, norms, and behaviour	Experience	Adaptation	Teamwork	Supportive culture
	Belief	Customer focus		
*Technical* Training and info support systems	IT systems	Training	Administrative support	Communication
	Competence			
*Strategic* Strategic importance and opportunity to change	Alignment	Resources	Physicians	Holistic approach
	Vision		Management	Continuous improvement
*Structural* Mechanisms to facilitate learning and disseminate best practices	External support	Accurate data	Staff involvement	Measurement
				System-wide scope

This framework suggests that the Lean CSFs and the context, content, application, and outcome of Lean should be viewed in light of the organisation’s strategic, technical, structural, and cultural environment [51]. Within the context (of the current situation and organisation), all three CSFs should be applied in view of the cultural, technical, strategic, and structural dimensions. The content of Lean interventions must be adapted to local conditions, with a focus on value creation for the patient, the culture of the workforce, substantial localised training on Lean tools and techniques, and accurate and robust data [51]. The collaboration of multi-skilled and multidisciplinary teams in the hospital, together with administrative project management, practical support, management and physicians’ engagement at the frontline, and the empowerment of staff, facilitates Lean application. Finally, a supportive environment with effective communication, feedback to employees and patients, the adoption of a holistic quality improvement philosophy, and the establishment of a long-term continuous improvement plan in a system-wide, multifaceted approach upholds the outcomes’ domain of the framework [51].

The three CSFs are ultimately identified and analysed, originating from the perceptions of senior managers working in public hospitals in KZN. The statistical methods and SEM fit indices presented above provide a basis for verifying the resemblance of these identified factors to the actuality at the population level.

## Conclusion

Applying EFA, CFA, and SEM, the study identified three critical success factors for the successful initiation of Lean in public hospitals in KwaZulu-Natal, South Africa. CSF1 (strategic leadership and organisational attitude), CSF2 (integration of Lean elements, tools, and techniques), and CSF3 (basic stability in operational processes) consist of key elements for managers to consider prior to the initiation of Lean.

Collaborative leadership and the embedding of Lean as a strategic driver is depicted in the literature as an important enabler of Lean in non-healthcare industries, and the study findings corroborate CSF1 as instrumental for Lean success also within the health care industry. The study uniquely identifies CSF2 and CSF3 as the other two critical success factors in KZN public hospitals, despite these being reflected in reviewed literature as having less importance in other studies. The application of the five Lean principles justifies these two CSFs as integral rudiments for successful Lean initiation in the healthcare industry.

Ultimately, each of the three CSFs cannot be viewed in isolation, as they all have significance in different dimensions of capability within the organisation. The use of these CSFs and the context, content, application, and outcome of Lean should be considered in view of the organisation’s strategic, technical, structural, and cultural environment. The three identified CSFs will form the basis for the development of the Lean Success Predictor for Rapid Initiation Tool (“Lean SPRInT”), which will be proposed as a Lean initiation and situational baseline assessment tool for public hospitals in KwaZulu-Natal, and also as universally applicable to South Africa at large.

The Lean SPRInT will be put forward as an initiation tool for managers to embark on the Lean transformation journey. Lean SPRInT uses sets of elements for the three CSFs for Lean implementation. Once rated by the user, these would yield a fuzzy logic output of graded Lean implementation readiness levels and processes that would guide managers to initiating Lean. The calculated Lean readiness levels, ranging from 1 to 3, for each of the CSF elements allows managers to gauge the deficiencies in their institution, which once improved, would predict greater success. In addition to the Lean SPRInT, Andersen et al.’s [51] framework suggests that Lean CSFs and the context, content, application, and outcome of Lean should be viewed in light of the organisation’s strategic, technical, structural, and cultural environment.

Finally and significantly, further research in the effectiveness of these CSFs for the introduction of Lean in South African hospitals will be beneficial to the Lean body of knowledge.

## Data Availability

Data extracted from completed questionnaires was collated onto an electronic spreadsheet. This collated data was used for statistical analysis and can be made available by the corresponding author upon request.
